# Pervasive Computing Technologies to Continuously Assess Alzheimer’s Disease Progression and Intervention Efficacy

**DOI:** 10.3389/fnagi.2015.00102

**Published:** 2015-06-10

**Authors:** Bayard E. Lyons, Daniel Austin, Adriana Seelye, Johanna Petersen, Jonathan Yeargers, Thomas Riley, Nicole Sharma, Nora Mattek, Katherine Wild, Hiroko Dodge, Jeffrey A. Kaye

**Affiliations:** ^1^Oregon Center for Aging and Technology, Oregon Health and Science University, Portland, OR, USA; ^2^Department of Neurology, Oregon Health and Science University, Portland, OR, USA; ^3^Department of Biomedical Engineering, Oregon Health and Science University, Portland, OR, USA; ^4^Neurology Service, Portland Veteran Affairs Medical Center, Portland, OR, USA

**Keywords:** in-home monitoring, technologies, smart home, sleep, gait, dementia, medication adherence, aging in place

## Abstract

Traditionally, assessment of functional and cognitive status of individuals with dementia occurs in brief clinic visits during which time clinicians extract a snapshot of recent changes in individuals’ health. Conventionally, this is done using various clinical assessment tools applied at the point of care and relies on patients’ and caregivers’ ability to accurately recall daily activity and trends in personal health. These practices suffer from the infrequency and generally short durations of visits. Since 2004, researchers at the Oregon Center for Aging and Technology (ORCATECH) at the Oregon Health and Science University have been working on developing technologies to transform this model. ORCATECH researchers have developed a system of continuous in-home monitoring using pervasive computing technologies that make it possible to more accurately track activities and behaviors and measure relevant intra-individual changes. We have installed a system of strategically placed sensors in over 480 homes and have been collecting data for up to 8 years. Using this continuous in-home monitoring system, ORCATECH researchers have collected data on multiple behaviors such as gait and mobility, sleep and activity patterns, medication adherence, and computer use. Patterns of intra-individual variation detected in each of these areas are used to predict outcomes such as low mood, loneliness, and cognitive function. These methods have the potential to improve the quality of patient health data and in turn patient care especially related to cognitive decline. Furthermore, the continuous real-world nature of the data may improve the efficiency and ecological validity of clinical intervention studies.

## Introduction

In the second half of the last century, medical practice became increasingly specialized. By the 1950s, the clinic or “doctor’s office” visit largely replaced the traditional home visit (Kao et al., 2009). The office visit in some respects affords greater convenience and efficiency for providers. For example, more patients can be seen in a shorter amount of time and patients can have direct access to an array of medical technologies. However, in other respects, it has resulted in patient inconveniences, inefficiencies, and potential inaccuracies in the assessment itself. Clinic visits are episodic in nature, occurring potentially once or twice a year with occasional “off schedule” appointments for unanticipated concerns. Visits average approximately 22 min (Cherry et al., 2008) and often occur at the point of a health crisis rather than at early onset of an illness. This has led to a more reactive than proactive approach to treating patients.

Another significant challenge to assessing an individual’s mental and functional status in the often annual office visit is that both patient self-report and collateral informant data are required, each of which might be biased or unreliable due to stress, worry, or forgetfulness (Wild et al., 2015). Given the implicit pressure to limit the duration of the visit, there is a high demand on patients to provide concise and well-organized health histories. This is a challenge particularly for individuals with Alzheimer’s disease and related dementias. Even, cognitively intact individuals may have difficulty remembering potentially important events or trends such as dietary habits, minor falls, medication changes, or sleep habits.

Most neurodegenerative diseases evolve slowly and initially affect function subtly, at first imperceptible to the patient and family until symptoms emerge that disrupt daily function. Mild cognitive impairment (MCI) is often considered a prodromal stage for a variety of neurodegenerative disorders including Alzheimer’s dementia and is used to describe individuals who have mild decline in their cognitive function but continue to engage independently in community and household affairs (Mariani et al., 2007). Subtle changes in a number of cognitively demanding daily activities (e.g., computer use, following a medication regimen) occur between normal cognitive aging, MCI, and dementia (Gold, 2012; Kaye et al., 2014; Schmitter-Edgecombe and Parsey, 2014). As these real-world functional changes are directly associated with cognitive changes, monitoring functional changes offers a means for early detection of incipient dementia. However, since the earliest changes are subtle and these individuals do not show frank impairments in these activities, these changes are often not brought to the attention of a patient’s doctor until later in the disease process if at all. Clinic visits in turn often occur only after cognitive decline has had an obvious impact on an individual’s functional abilities, rather than earlier in the disease course when interventions may delay decline.

Finally, clinic visits are inconvenient for many patients, requiring travel from their homes, sometimes over long distances. This may be especially challenging for elderly patients, particularly those who live in rural areas, have stopped driving, or have physical disabilities making it difficult to move around outside the home. Over 50% of all patients with dementia go undiagnosed by their primary care provider (Boise et al., 1999, 2004; Bradford et al., 2009; Kotagal et al., 2015), which may be due in part to the limitations of conventional approaches to cognitive and functional assessment noted above.

Researchers at Oregon Health and Science University (OHSU) Oregon Center for Aging and Technology (ORCATECH) have established an approach to transform the clinical assessment first in a research setting and then ultimately within the clinical enterprise. ORCATECH researchers have designed and are using a “smart home” system to carry out research. Interest in “smart home” research has grown over the last couple of decades as a means for improving patient health and independence. “Smart home” research covers an array of approaches for collecting data continuously on in-home activity. A number of technologies have been employed. These have included either smart home environmental sensors [video, passive infrared (IR), contact, pressure sensors] or the use of wearable technology (Cook and Krishnan, 2014). Continuous in-home monitoring and analysis of these data enable creation of better models of human behavior and prediction of changes in health. For example, researchers have used smart home data to give older adults feedback on their functional abilities (Lee and Dey, 2010), to correlate changes in how participants made coffee and changes in performance on neuropsychological tests (Hodges et al., 2010), to group participants into cognitive health categories (Dawadi et al., 2013), and to detect health status decline (Rantz et al., 2008, 2009).

The approach taken by ORCATECH has been to fully outfit homes with an unobtrusive platform of in-home sensors to be able to continuously collect daily activity data on hundreds of research volunteers. The activity data collected continuously over months and years enables the detection of changes in individual function around key behaviors relevant to change in cognitive status. These data provide the foundation for the development of predictive models relating changes in functional status to ongoing and future cognitive decline. Furthermore, this research has led to the development of new technologies to collect real-time data continuously and unobtrusively. All of this work has the potential to strengthen the ability of families and clinicians to be proactive in treating health in general – and cognitive decline in particular – without relying solely on the episodic or annual clinic visit. We present a review of the ORCATECH research initiative as it has developed over the last decade. After reviewing our research objectives, we describe the sensor platform installed in each home and provide an overview of exemplary studies that have benefited from ORCATECH’s continuous data collection.

## Research Objectives

ORCATECH’s research is focused around several common goals. Our first goal has been to develop unobtrusive and continuous monitoring platforms, along with novel algorithms and assessment techniques for detecting motor and cognitive change. Second, we now seek to determine if these developed algorithms and techniques can be used to detect early or prodromal cognitive decline in older adults living in typical community settings. Third, we are focusing on identifying how these technologies might be best adapted for daily care practice to enhance the monitoring needs and optimize communication between patients and health care professionals. Finally, our goal is to make the system available for developing new interventions and conducting vitally needed clinical trials to advance dementia management and treatment.

## Method

### Volunteer recruitment

Beginning in 2007, ORCATECH began scaled deployment of a simple home-based platform of ubiquitous sensors in hundreds of homes of older adults living independently in the community (see Kaye et al., 2011). Participants were all recruited from the Portland metropolitan area, including older adults living in retirement communities as well as free-standing single-family homes. Potential participant pools were created following formal community presentations. Participants were also recruited from lists of current OHSU Layton Aging and Alzheimer’s Disease Center research participants who have been followed longitudinally in other projects.

All of the volunteers recruited met the following inclusion criteria: they were 70 years or older and living alone or with their spouse or partner (not as a caregiver). Volunteers with a Mini-Mental State Examination score >24, a Clinical Dementia Rating scale score ≤0.5 and a physical examination demonstrating average health for age were invited to participate. While these were the inclusion criteria used in the initial screening, changes in the participants’ status after enrollment on any one of these criteria would not lead to exclusion from the study. The OHSU Institutional Review Board approved all of the research studies summarized below. All participants provided written informed consent.

Participation required filling out an initial comprehensive base line survey as well as a weekly brief online life events and health questionnaire. Participants also agreed to be continuously monitored through a platform of sensors to be installed in their homes. Older adults participating in the study were already computer literate or became computer literate through training provided by the Center. Computer literacy was defined as being able to reliably send and receive email. Computer literacy ensures that participants are able to complete a weekly health form and can communicate with research staff directly online. Participants who did not already have a personal computer were given a computer in order to be able to complete the weekly survey as well as Internet access if they did not have it. The provided computer could also be used at their leisure for general use as well.

### ORCATECH platform design

Each participant’s home was outfitted with several types of sensors – wireless passive IR motion sensors (MS16A; http://X10.com) strategically placed in each room and wireless magnetic contact sensors (DS10A, http://X10.com) placed on the outside doors and the refrigerator. Upgrades to this system have included IR sensors operating via the Zigbee wireless communication protocol (NYCE Control). The personal computer each participant received served as another type of sensor. General computer use (e.g., time on computer, mouse movements) comprised data collected via this activity. Based on specific research needs, some homes received additional sensors. These included medication trackers (Hayes et al., 2006), phone monitors (Shenzen Fiho Electronic, Fi3001B), and a wireless scale (Withings) capable of measuring body mass index, weight, pulse, temperature, and air quality.

**Figure 1:**
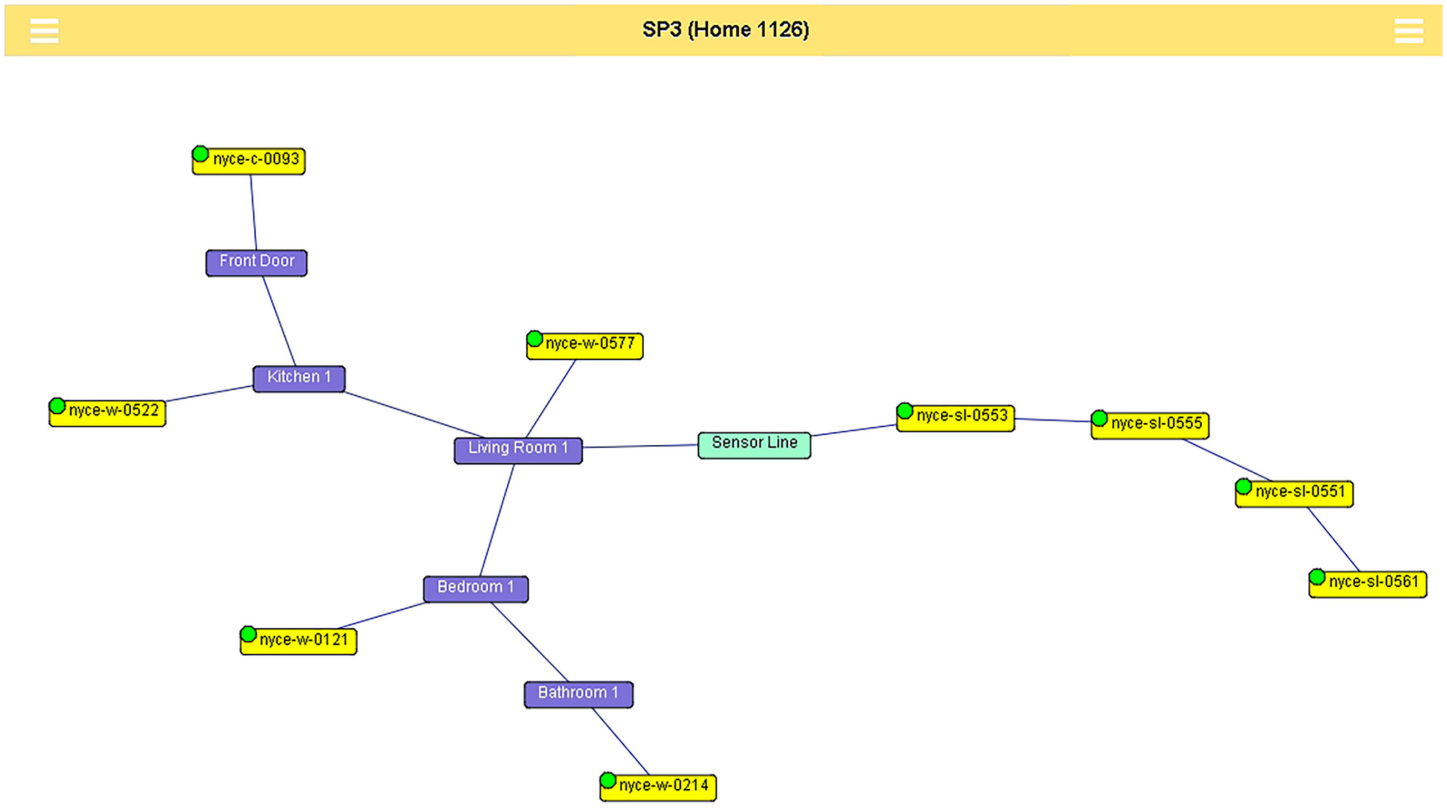
**Each room in a home is linked to the others based on valid room transitions**. Passive infrared sensors are linked to the rooms in which they are located. The walking sensor line is an area, consisting of four restricted field passive infrared sensors linked together in the order in which they are placed. The walking sensor line links room(s) in which the sensors are located. Green or red dots on the sensor nodes indicate if the sensor is currently reporting in to the sensor network.

Whenever they detect motion, the motion sensors send a signal wirelessly to a small computer (GlobalScale Dreamplug) placed out of the way in the participant’s home. The contact sensors send event-based codes whenever a door is opened or closed (See Figure 1 for a schematic depiction of how these sensors might be interconnected in a home.). Additionally, the contact sensors send a “heartbeat” every hour, which ensures proper functioning of the device. Data collected from the various sensors and the personal computer are sent nightly over a broadband connection and is stored in a SQL database on secure research servers and managed by custom software.

By fusing data from these various sensors, time series data are constructed regarding when and where activity is taking place in the home. Using these time series, profiles of fluctuations in-home activity including when a participant has exited and reentered the home are created. Because data are collected for a given individual over the course of months to years, accumulated data provide a longitudinal profile of changes in activity for analysis and interpretation. With these data, predictive tools for detecting cognitive and functional change are developed. In the following, we highlight examples of how these data have been used to identify meaningful activity and outcomes relevant to aging, cognition, and function.

## Research Evidence

Through continuous in-home monitoring, the ORCATECH group has investigated a range of quantifiable activities that may reflect changes in cognitive status. These have included sleep, computer use, medication adherence, walking speed, overall activity patterns, and time out-of-home. Most of these overlap with and are not dissimilar from the type of activities assessed as either activities of daily living (ADLs) or instrumental activities of daily living (IADLs). The assessment of ADLs and IADLs has been used in geriatrics and dementia care as a tool for guiding decisions to move from independence to higher levels of care. There has also been recent interest in examining the frequency and types of IADL difficulties that occur in normal aging and MCI to inform development of targeted interventions for IADL impairment (Seelye et al., 2013). In the following sections, we offer examples of how we have collected and analyzed data in each of these functional domains through the technology platform installed in homes. In each case, we highlight the potential for continuous in-home monitoring to improve understanding of functional, motor, and cognitive status and to predict cognitive change.

### Sleep

Disrupted sleep is a significant problem among older adults and is associated with a number of other health-related problems including cardiovascular and pulmonary co-morbidities, decreased quality of life, and increased fall risk. In addition, there is evidence demonstrating a correlation between sleep disturbance and Alzheimer’s disease or MCI (Lyketsos et al., 2002; Tractenberg et al., 2005; Tworoger et al., 2006). Previous research has also demonstrated a correlation between disrupted sleep, sleep latency, and wake after sleep onset (WASO), with performance on memory tests the following day for individuals with amnestic MCI (aMCI) (Westerberg et al., 2010) as well as cognitively intact elderly (Seelye et al., 2015a). One of the goals of ORCATECH has been to better understand the relationship between sleep and cognitive decline through continuous assessment of sleep behavior over an extended period of time.

In keeping with the goal of long-term, naturalistic assessment, the ORCATECH platform focuses on passive sensing of bed and bedroom activity to differentiate a number of relevant sleep parameters. The passive sensing approach has been validated to the ground truth of pressure mats in bed as well as showing favorable comparison to wrist worn actigraphy. As with all movement-based estimates of sleep measures (including actigraphy and bed mats), variables such as total sleep time (TST) must be inferred from periods of inactivity (Hayes et al., 2010). A validated algorithm was used to derive these measures against the ground truth measure of movement on the bed. Because data were obtained over a long-time span (26 weeks), this allowed for the data to be analyzed for central tendencies and variability while accounting for episodic activity outliers that may skew the data (e.g., up more frequently at night due to illness, increased restlessness due to unusual levels of daytime activity). These data, collected continuously over 6 months, provide information about an individual’s “typical” night rather than a single night as in a polysomnography study, for example.

Using this approach, ORCATECH researchers collected objective sleep measures over an extended period of time comparing cognitively intact, aMCI, and non-amnestic MCI (naMCI) volunteers (Hayes et al., 2014). They found that aMCI volunteers had less disturbed sleep than both naMCI and cognitively intact volunteers, and in general, the naMCI volunteers showed a level of disturbed sleep that was intermediate to that of aMCI and intact volunteers. These differences contradicted self-report data that had suggested no differences in sleep behaviors across groups, underscoring the value of collecting frequent and objective in-home measurements. In contrast to some past studies (Geda et al., 2004), our findings suggest that aMCI volunteers typically experience less sleep disruption during the night than cognitively intact volunteers.

Another sleep study examined the relationship between episodes of poor sleep and cognitive test performance in cognitively intact older adults (Seelye et al., 2015a). Specifically, researchers examined the impact of sleep disturbance and sleep duration the *night prior*, the *week prior*, and the *month prior* to cognitive testing performance in the clinic. Results showed that more sustained periods of disturbed sleep, i.e., mildly disturbed sleep the week prior and month prior to cognitive testing was associated with reduced working memory on cognitive evaluation. On the other hand, one night of mild sleep disturbance was not associated with decreased cognitive performance the next day. Sleep duration was unrelated to cognitive test performance.

These findings suggest that in-home, unobtrusive sensor monitoring technologies provide a novel method for objective, long-term, and continuous assessment of sleep behavior and other lifestyle factors that might contribute to decreased or variable cognitive performance in healthy older adults. Future longitudinal studies of normal aging and MCI using sensor-based sleep assessment will explore whether mild sleep disturbance contributes to intra-individual variability in cognitive test scores over time.

### Computer Use

Computer use is becoming increasingly ubiquitous among older adults. Currently, 59% of those 64 years or older go online and this number is increasing at a rapid pace (Smith, 2014). This is significant when considering cognitive health. Computers offer older adults the option of maintaining connections with a larger social network without leaving their homes. This has potential benefits to emotional and psychological health. Computers also provide a means for retrieving and storing important health information, and for exchanging health information directly with health care providers. All of these applications could help older adults remain independent in their homes longer or find help when cognition and function become a challenge. Finally, computer use is a complex task taxing multiple cognitive domains (attention, working memory, episodic memory, executive function, etc.) and an individual’s use patterns – or changes in these patterns – may signal important cognitive changes. For example, the frequency and duration of computer use have the potential for offering insight into changes in cognitive function among the growing aging population.

An important part of the ORCATECH approach has been to ensure that when possible all research volunteers have access and could use a computer even at a very basic level. One of the volunteers’ primary research tasks is to complete a weekly online survey using either their own computer or tablet or a laptop computer provided by the Center. The weekly online surveys have been important to the data collection as they provide information on activities that are difficult to infer by passive sensing alone (e.g., reports of pain, mood, falls, etc.). Additionally, the computer serves as an additional activity and behavior sensor, providing an opportunity to assess computer use on a specific task (completing the questionnaire) at a minimum of once a week in addition to the person’s typical computer-based activity.

Custom-designed computer monitoring software (CMS) is installed on each of the computers. When participants use the computer, the CMS software requires them to log in, which in turn initiates computer use monitoring (Hatt et al., 2009; Kaye et al., 2014). Multiple types of events may be recorded from the participants’ computers (general typing data, login events, login passwords, application focus change events, and mouse events). For example, keyboard typing is recorded in sets of three keystrokes, called *trigrams*, with the amount of time elapsed between the three key press events. The trigram is also time stamped with a 1 hour resolution, providing researchers with information about the approximate time of computer use and how fast the user was typing. Recording is done when a participant is typing a document or an email. Using trigrams of general typing data provides quantifiable information on typing strokes in terms of speed and duration while at the same time making it possible to respect individual privacy by restricting and obfuscating information about actual content (Hatt et al., 2009; Pavel et al., 2010).

ORCATECH researchers have used the above techniques to evaluate the relationship between computer use and cognitive decline. In one study, researchers collected data on 230,000 computer sessions over an average of 36 months from 113 computer users (mean age, 85 years; 38 with MCI) (Kaye et al., 2014). These data were assessed to compare computer use over time between individuals with and without MCI. Computer sessions were described by mouse movement data. Each mouse movement of more than five pixels generated a Windows event that was saved and time-stamped. In order to remove slow drifts in mouse position during inactivity, the computer was considered to be in use only when there were more than 100 mouse events within a 5-min period. Additionally, days of computer use were counted as days when a 5-min period was recorded. Mean daily usage (in hours) was the sum of total time on the computer per month divided by total number of days with usage in the month. Consistency in day-to-day computer use was indexed using the coefficient of variation.

This study was the first unobtrusive in-home monitoring and analysis of computer use in an aging and MCI population. While at baseline, there were no differences in use metrics between the two groups (likely reflecting initial training), over time there was a decrease in number of days per month, mean daily use, and day to day variability of use among participants with MCI as compared to cognitively intact participants (Kaye et al., 2014). In a survey of attitudes and beliefs about their computer use, MCI volunteers reported less confidence and more anxiety over time while using their computer relative to cognitively intact older adults, who gained confidence over time (Wild et al., 2012). These studies suggest that continuous assessment of computer use may be an efficient and sensitive method to evaluate early decline among individuals with MCI.

Another computer-based study (Austin et al., 2011c) investigated the relationship between motor speed and typing speed on a computer keyboard during computer login events. These login events consisted of typing a user name and password at the beginning of each participant’s regular computer use session. The speed of typing was measured by inter keystroke intervals (IKI), which is the time in between consecutive keystrokes calculated from the trigrams. Motor speed was assessed by the Halstead–Reitan Finger Tapping Test (FTT) (Strauss et al., 2006). Data were analyzed from 22 participants who used their computer frequently in the 28-day period surrounding an annual neuropsychological exam. This study found a high correlation between tapping speed and typing with the dominant (*r* = 0.7, *p* < 0.0002) and non-dominant (*r* = 0.77, *p* < 0.0001) hand. One conclusion from this study is that monitoring speed of typing over time during regular computer use could unobtrusively detect motor decline known to precede cognitive impairment (Camicioli et al., 1998).

Ongoing studies are examining how other aspects of remotely monitored home computer use might be related to early cognitive changes in older adults. For example, preliminary work suggests that aspects of responding to the weekly online health questionnaire differ among cognitively intact older adults and those with MCI over 1 year (Seelye et al., 2015b). Computer mouse movements obtained during routine, everyday home computer use are also being examined as a novel method to identify real-world cognitive changes that are associated with MCI in older adults.

This research suggests that computers provide an additional sensor environment for investigating the relationship between functional change and cognitive change. Successful computer engagement not only requires intact complex motor and cognitive skills but also facilitates social connections. In the final section, we will discuss how computers used for online video chat may help us better understand the relationship between functional changes in social interaction and cognitive decline.

### Medication adherence

One of the greatest challenges facing persons with AD and other dementias is adherence to medication regimens. More than 76% of Americans aged 60 years and older take two or more prescription medications (Gu et al., 2010). Forgetful patients are inconsistent in their adherence and tend to be poor historians of when and how often they are taking their medications. Medication non-adherence leads to increased risk of hospitalization and mortality among the older population and in those with chronic diseases (Col et al., 1990; Ho et al., 2006).

Medication adherence relies on a set of cognitive skills, including prospective memory and executive functioning. Activities dependent on memory and executive function tend to be most difficult for older adults with MCI (Brooks, 2006; Allaire et al., 2009; Schmitter-Edgecombe et al., 2009; Griffith et al., 2010). Thus, adherence to a medication regimen can be an everyday task that if monitored can act as a bellwether of changes in cognitive function. Additionally, a system that independently monitors medication adherence can alert health care providers and informal care providers in real time. ORCATECH researchers have used medication-taking behavior and adherence data to develop systems to facilitate improved patient adherence and medication usage (Lundell et al., 2007; Hayes et al., 2009a,b; Pavel et al., 2010).

Insel and colleagues reported that a composite of executive function and working memory was a significant predictor of reduced medication adherence in a population of older adults (Insel et al., 2006). Building on this observation, ORCATECH researchers examined whether objective evidence of an individual’s difficulty with medication adherence could identify those with early cognitive impairment. Using a 7-day electronic pillbox called the MedTracker, which records the time of day when a day’s compartment is opened (Hayes et al., 2006), a 5-week drug adherence trial using vitamin C supplements was conducted. Participants took this supplement from the MedTracker twice daily at pre-specified times approximately 12 h apart ensuring that each person faced the same daily challenge. Adherence was calculated in two ways. First, there was overall adherence calculated as the percentage of days when both pills were taken. Second, regimen adherence was calculated by the percentage of times the volunteer took their medication within a window of 1 h before to 2 h after the prescribed times. An individual was considered to have poor adherence to the regimen if their adherence to one of the two measures was <80% (Hayes et al., 2009a).

Participants, who were all deemed cognitively intact at the time of the study, were divided into a relatively lower cognitively performing group compared to a higher performing group (HPG) based on their total score at entry on the Alzheimer’s Disease Assessment Scale Cognitive Subtest (ADAS-cog). The cognitive differences between the groups were slight [mean ADAS-cog score in the lower performing group (LPG) was 10/40 vs. 6/40 in the HPG; lower score indicating better performance]. Despite this subtle distinction, there was a significant difference observed in medication adherence between the two groups with the LPG having significantly poorer total adherence than the HPG (LPG: 63.9 ± 11.2% adherence, HPG: 86.8 ± 4.3%). There was a 4.1 relative risk of non-adherence in the LPG as compared to the HPG. This study provides strong evidence that even very MCI in healthy older individuals has a detrimental impact on medication adherence and has important implications for clinical trials with MCI patients (Hayes et al., 2009a,b).

### Movement patterns and walking

Almost two decades ago, motor slowing was demonstrated to precede the diagnosis of dementia shown by conventional in-person timed tests (e.g., using a stop-watch) (Camicioli et al., 1998). It is based on this research and others that ORCATECH researchers have sought to study both movement patterns and variation in walking speeds using continuous in-home measurement.

#### Mobility and Walking Speed

Walking speed is typically measured during a clinic visit. Some of the methods used to assess walking speed include using a stop-watch and counting steps, using a gait mat or by wearing an accelerometer. However, with the traditionally episodic nature of clinical evaluations and gait measurement, it is difficult to distinguish between abrupt changes in function and changes that occur more slowly over time. Body-worn accelerometers, which typically assess gait speed by identifying footfalls, are an alternative to clinic assessments as they can be used to continuously assess walking speed wherever a participant goes. However, remembering to wear or charge such devices can be challenging, especially among a memory impaired, older adult population. In addition, current accelerometry approaches do not provide location of the detected activity within a home. Furthermore, walking in the home environment is generally of low velocity and rarely linear or sustained, making the algorithms used to detect “walking” with accelerometry difficult to interpret. Among older adults in particular, it is therefore important to apply ecologically valid techniques to *unobtrusively* assess important metrics such as walking speed. The ability to continuously assess walking speed in the home provides a better understanding of the variability and change in walking speed of an individual over time.

To that end, we developed a method to assess walking speed using an array of in-home sensors. With careful in-series placement of wireless IR sensors in the home, walking data can be passively captured that identifies how quickly and frequently participants are passing by this sensor line on a daily basis (Hayes et al., 2008; Hagler et al., 2010; Austin et al., 2011b; Kaye et al., 2012). Algorithms estimating the speed of walking from the in-home sensor data have been validated against a “gold standard” gait mat (Hagler et al., 2010). This provides a more dynamic result than can be gained from a single assessment in the clinic or the home.

To examine how total activity during the day and walking speed more specifically might differentiate older adults with MCI compared to those with intact cognitive function, we captured over 108,000 person-hours of continuous activity data for up to 418 days (mean 315 ± 82 days) using the unobtrusive sensor system in the homes of 14 elderly volunteers (Hayes et al., 2008). In addition to mean or median measures of walking speed and amount of activity in the home, wavelet analysis was used to examine variance in activity at multiple timescales. The coefficient of variation in the median walking speed was twice as high in the MCI group as compared to the healthy group. Furthermore, the 24-h wavelet variance was greater in the MCI group (MCI: 4.07 ± 0.14, healthy elderly: 3.79 ± 0.23; *p* = <0.008) indicating that the day-to-day pattern of activity of subjects in the MCI group was more variable than that of the cognitively healthy older adults (Hayes et al., 2008).

In a study of 76 older adult volunteers living alone, measurements of walking speed were taken throughout the day for a 1-month period. These subjects generated a total of 39,474 walking episodes, which equated to 500 walks per subject per month. Holding constant age, sex, education, and Geriatric Depression Scale score, we found that a 1 SD increase in the global cognition *z*-score was associated with a 10.1 cm/s increase in mean in-home walking speed. Similarly, a 1 SD increase in attention *z*-score corresponded to a 7.7 cm/s increase in walking speed (Kaye et al., 2012).

In another ORCATECH study (Dodge et al., 2012), walking speeds were evaluated in 54 participants with intact cognition, 31 participants with naMCI, and 8 participants with aMCI at baseline, with a mean follow-up period of 3 years. Latent trajectory models identified three distinct trajectories (fast, moderate, and slow) of mean weekly walking speed. Participants with naMCI were significantly more likely to be in the slow speed group than in the fast or moderate speed groups. Further, there were four distinct trajectories of change with regard to variability in walking speed over time (measured by the coefficient of variation, COV): group 1, the highest baseline speed with increasing COV followed by a sharply declining COV; groups 2 and 3, relatively stable speeds and COV; and group 4, the lowest baseline and decreasing COV. Participants with naMCI were significantly more likely to be members of either highest or lowest baseline COV groups (groups 1 or 4), possibly representing the trajectory of walking speed variability for early- and late-stage MCI, respectively. Thus, walking speed and its daily variability may be taken as an early marker of the development of MCI. Patterns of movement and mobility turn out to be another.

#### Patterns of Movement and Mobility

Interpretation of data from remote monitoring of activity relies on the assumption that an individual’s activities fall into patterns of varying predictability and once measured and documented, deviations from their established patterns provide the opportunity to detect meaningful change. However, it should be noted this does not assume that the opposite is necessarily true, i.e., that a person with no changes in routine patterns of activities is not experiencing cognitive decline. It may be that an individual with AD or another dementia may engage in very stereotyped behaviors to hide or compensate for cognitive decline. An important goal of ORCATECH research has been to better understand the relationship between activity patterns and cognitive decline.

Both establishing individual activity patterns and measuring meaningful change in these patterns pose a significant challenge. Traditionally, data gathered on mobility have focused on out of home mobility, such as for the purpose of tracking epidemics or planning transportation, and have demonstrated predictable patterns of mobility using time-independent universal scaling laws. Studying a data set of almost 15 million observations from 19 adults spanning up to 5 years of unobtrusive longitudinal home activity monitoring, we found two main results. We first found that the universal scaling laws shown to describe mobility outside of the home (González et al., 2008) did not hold for mobility in the home. Second, we found that there was substantial predictability and regularity in in-home mobility when accounting for the context of the individual movements (Austin et al., 2014a). This investigation focused on the temporal regularity and predictability of the number of times an individual moves between different rooms in their home. We found that like out of home mobility, in-home mobility is also highly stereotyped, albeit in a different way, which may have applications for predicting individual human health (Campbell et al., 2011) and functional status (Evans et al., 2011; Kaye et al., 2012) by detecting adverse events or trends (Candia et al., 2008) and in conducting more meaningful clinical trials (Carlsson, 2008; Kaye, 2008).

### Social Engagement

Research has demonstrated that higher levels of social engagement can be protective against cognitive decline (Fratiglioni et al., 2000; Scarmeas et al., 2001; Wang et al., 2002; Wilson et al., 2002; Akbaraly et al., 2009). Individuals who are more socially active generally exhibit numerous positive health benefits including higher self-rated health (Cornwell and Waite, 2009) and lower all-cause mortality (House et al., 1988; Glass et al., 1999; Berkman et al., 2000; Holt-Lunstad et al., 2010). Additionally, there has been research to demonstrate that even brief social interactions lasting a few minutes can lead to increases in performance on subsequent tests of executive function (Ybarra et al., 2008).

Better understanding of the relationship between social engagement and cognitive decline opens the possibility for both early identification of those at risk of cognitive decline and development of prevention strategies using social engagement as a tool to improve cognitive function. Research in this area has begun using the in-home technologies described above as well as developing new data collection methods (Dodge et al., 2014; Petersen et al., 2014a). Several aspects of social engagement may be assessed passively. These include time out of the home (time with the “outside” world), amount of telephone use, frequency and duration of visitors to the home, and time on the Internet spent in certain activities (e.g., email, video chat). In the following, we highlight some of these preliminary investigations related to both identification and intervention of cognitive decline including evaluations of time spent out of house, telephone usage, use of Internet-based video chat, and the contribution of social engagement to risk of being placed in a long-term care (LTC) facility.

#### Time Out of Home

Spending time outside the home is a complex activity, requiring navigation, wayfinding, and physical capability (Wahl et al., 2013). Individuals with MCI have been shown to not only spend less overall time out of the home (Suzuki and Murase, 2010) but also do not travel as far from the home compared to healthy controls (Crowe et al., 2008; James et al., 2011; Shoval et al., 2011; Wettstein et al., 2015). These differences may be due to the cognitive demands involved in leaving the home. Additionally, leaving the home is important for maintaining quality of life, independence, and health in older adults (Gagliardi et al., 2010). Given that typical healthy older adults spend an average of about 20 h in their home per day (Kaye et al., 2011), the amount of time out of the home becomes particularly salient in evaluating reduced direct social engagement. Most of the techniques to assess time out-of-home to date depend on subjective self-report, for example, life space analysis (Crowe et al., 2008; James et al., 2011). However, such techniques tend to suffer from bias by recency effects, and cannot be maintained long-term.

Recently, Global Positioning Systems (GPS) have been employed to assess not only total time spent outside the home but also locations visited and walking speed while out (Shoval et al., 2011; Wahl et al., 2013; Wettstein et al., 2015). However, much like accelerometers to assess walking speed, this approach does not work well for older adults, especially those with memory complaints, as it depends on the adult to remember to carry or wear the device. To mitigate these shortcomings, we developed a technique to assess time out-of-home unobtrusively and continuously. In this technique, time out of the home was identified using a logistic regression classifier that takes into account firing of sensors within specific home locations as well as at the exit door(s). This approach was both sensitive (0.939) and specific (0.975) in detecting time out-of-home across over 41,000 epochs of data collected from four subjects monitored for at least 30 days each in their own homes. The method has the advantage of not only detecting the total daily hours spent outside the home but also the time of day the participant was out of the home. By being able to track not only the duration of time spent outside the home each day but also the time of day participants were out of the home, general behavioral patterns of older people could be documented. We found on average that half of the participants were outside their homes at noon and 6:00 p.m., likely corresponding to lunch and dinner hours. Additionally, we were able to show that higher time out-of-home is associated with lower levels of loneliness, as measured using the UCLA Loneliness Scale (Russell et al., 1980), and with higher levels of self-reported physical activity, using the physical activity component of the Berkman Social Disengagement Index (Petersen et al., 2014a).

Time out of home has also been shown to relate to relevant behavioral and psychological changes that may be commonly experienced by MCI or dementia patients such as low mood. Thus, using 18,960 weekly observations of online reported mood ratings over a period of 120 weeks, it was found that during weeks of low mood, participants spent less time out of residence, but did not show changes in walking speed or movement about their home measured by room transitions (Thielke et al., 2014).

#### Telephone Usage

Because people use the phone to call their network of friends, family, and acquaintances, monitoring phone use could give a picture of the size of the network (by monitoring numbers dialed) and the frequency of contact with the network (by monitoring total number of calls). An extensive body of literature has used mobile phone apps to detect and analyze the social network characteristics of younger individuals (Eagle and (Sandy) Pentland, 2006); Palla et al., 2007). However, older adults, especially the oldest old (85 and older) who are most susceptible to cognitive decline, have been slow to adopt mobile phone technologies. This means that landline use must be accounted for in this population.

Using phone monitors (Shenzen Fiho Electronic, Fi3001B) installed in the home, we have developed a technique to monitor landline phone use. These monitors work by detecting signals on the phone corresponding to phone events including “on-hook,” “off-hook,” number dialed, and “ring start.” Using data from the monitors installed in the homes of 26 older adults for 6 months, we have shown that overall phone use is negatively associated with cognition (Petersen et al., 2014b).

Recording telephone conversations and analyzing their content have been another avenue of exploration carried out by ORCATECH collaborators (Stark et al., 2014). In this study by using standard natural language processing techniques to analyze incoming and outgoing calls from a few homes, researchers were able to differentiate business from personal calls, family from non-family calls, familiar from unfamiliar calls, and family from other personal calls ranging from 74 to 88% accuracy. Other groups have experimented with recording and analyzing facial expressions through video monitoring to infer emotional status (Hosseini and Krechowec, 2004). One limitation to this approach, however, is that it can be considered intrusive given the video data that are collected. ORCATECH researchers and collaborators are continuing to explore ways that social relationships and changes in mood may be inferred remotely and unobtrusively by analyzing speech and activity patterns.

#### Visitors

Another important aspect of evaluating social engagement is visitors to the home. When striving to be completely unobtrusive (not requiring subjects to wear any devices) and anonymous (contain no information about who is causing the sensors to fire), the presence of a visitor in the home cannot be directly extracted from in-home sensor data. As a first approach to identifying visitors using this anonymous sensor data, we developed a classifier to distinguish times when visitors were present in the home from times when they were absent using key features from the in-home motion sensor data (Petersen et al., 2012). For each 15-min interval, we calculated the dwell time in key living spaces (living room, dining room, kitchen, and bathroom), total number of sensor firings, and number of transitions between the major living spaces. Then, using twice daily diary-based (over 6 weeks) self-report from two subjects as ground truth, ORCATECH researchers trained and tested a Support Vector Machine classifier, which was able to detect the presence of visitors in the home with a sensitivity and specificity of 0.90 and 0.89 for subject 1 (with 31.8 h with visitors), and sensitivity and specificity of 0.67 and 0.78 for subject 2 (with 89.5 h with visitors). These preliminary data not only demonstrate the feasibility of detecting visitors with anonymous in-home sensor data but also highlight the need for more advanced modeling techniques, so the model performs well for all subjects and all types of visitors.

#### Online Video Chat

ORCATECH researchers have also investigated changes in the quantity of social interaction and its effects on cognitive function in a randomized controlled clinical trial (RCT). This proof of concept RCT examined whether stimulation through social interaction using contemporary communication technologies (PC, webcams, and Internet) could improve cognitive function (Dodge et al., 2014). The RCT participants with normal cognition or MCI were engaged daily by trained interviewers or “conversationalists” via the Internet and PC webcams to facilitate participating in 30–40 min of standardized, semi-structured conversations for 6 weeks. The control group received only a weekly phone call to monitor the amount of social interaction. In addition to measuring cognitive function by traditional and computerized cognitive tests, the trial assessed whether an increase in daily conversation leads to changes in emotional well-being (secondary outcome) and speech characteristics (exploratory outcome) such as word counts, characteristics of words used, proportion of filler words, and sentence complexity.

Several methodological innovations for delivering social interactions were tested in this trial. Specific software was developed, so older adults who may not have had any experience with computers could answer calls by simply touching the screen of the monitor instead of using a mouse or keyboard. The daily interview sessions were automatically recorded. Automated speech-recognition algorithms were refined to examine the speech characteristics associated with cognitive status and their changes over time. Additionally, participants wore small digital micro-recorders to track the time and duration of conversations occurring outside of the online chat session. In the RCT, the 83 participants (mean age 81 years) who met the study inclusion criteria and were randomized to the trial showed exceptional adherence to the protocol. There was no dropout and the mean percent of days completed out of the total possible sessions was 89. The intervention group improved on an executive/verbal fluency cognitive measure (*p* = 0.02) (Dodge et al., 2015).

Also examined was whether speech characteristics differ by cognitive status (MCI vs. intact cognition). Using the recorded conversation during intervention sessions, MCI participants were found to speak more words (i.e., had a higher proportion of words generated out of total word counts) compared with those with intact cognition (Kaye et al., 2014) There are several possible explanations for this finding: first, MCI participants may be more likely to need to substitute words in the conversation to convey their thoughts, especially in the early stage of MCI when phonemic fluency is still preserved, leading to an increased proportion of speech duration in timed conversations. Second, individuals with MCI may have subtle difficulties with executive and self-monitoring aspects of conversation including reduced passage-of-time estimation abilities relative to those with normal cognitive function. Third, individuals with MCI may acquire deficits in social cognition, i.e., misreading of social cues and intent during social exchanges that could result in more discursive conversation among MCI participants. Importantly, this metric appeared to discriminate participants with MCI from intact participants better than the traditional cognitive tests of Animal Fluency and CERAD Word List Delayed Recall.

### A Multi-Domain Approach

In conventional model building a relatively restricted range of factors (e.g., demographics, baseline clinical measures) have been used to predict salient outcomes for individuals at risk for dementia. In order to build more accurate and meaningful models for assessment and intervention in MCI and dementia, integration of multiple data types and streams captured from the ubiquitous in-home computing platforms is needed.

Toward this end, an initial study using the conventional clinical measures typically used to predict a high value outcome – being placed into LTC was developed. In this study, ORCATECH researchers used a multi-factorial analysis combining data on risk factors for cognitive decline (social activity, sleep disturbances, and depressive symptoms) and the role they played in the risk of an older adult being placed in LTC (Miller et al., 2014). Controlling for cognitive and functional impairment, age, and medical conditions, each unit increase in social activity was associated with a 24% decrease in the risk of placement [odds ratio (OR) = 0.763, *p* = 0.001, 95% confidence interval (CI) (0.65, 0.89)]. While cognitive impairment, medical conditions, and age were also significant individual predictors of placement, many of these risk factors for placement are not readily modifiable. However, older adults who engage in more social activity outside the home may be able to delay transition from independent living (Miller et al., 2014).

Using the same cohort, but applying a fusion of variables from multiple assessment domains including the continuous sensed data, it was found that this approach could dramatically improve the prediction of care transitions to higher levels of care within 6 months. This study included 108 participants living alone who were followed longitudinally between July 2011 and March 2014. The mean age at study enrollment was 81.5 years (SD = 6.6 years); 89 participants were female (82%), and 81 were white (75%) (Austin et al., 2014a). During the monitoring period, 12 participants (11%) transitioned out of independence. In-home monitoring variables sampled at 1 min intervals were combined with clinical assessment, weekly self-report, and demographics/co-morbidities in a longitudinal logistic regression (Austin et al., 2014b). There were a total of 66,172,380 total samples collected during the monitoring period on a total of 34 covariates including the model constant. Thirty-six variables may seem high for only 108 participants, but only five of the variables – age, number of rooms in the home, years of education, socioeconomic status, and residence – were constant during the monitoring period. The remaining variables changed over time and were hypothesized to be predictive of a future transition to a higher level of care.

**Figure 2:**
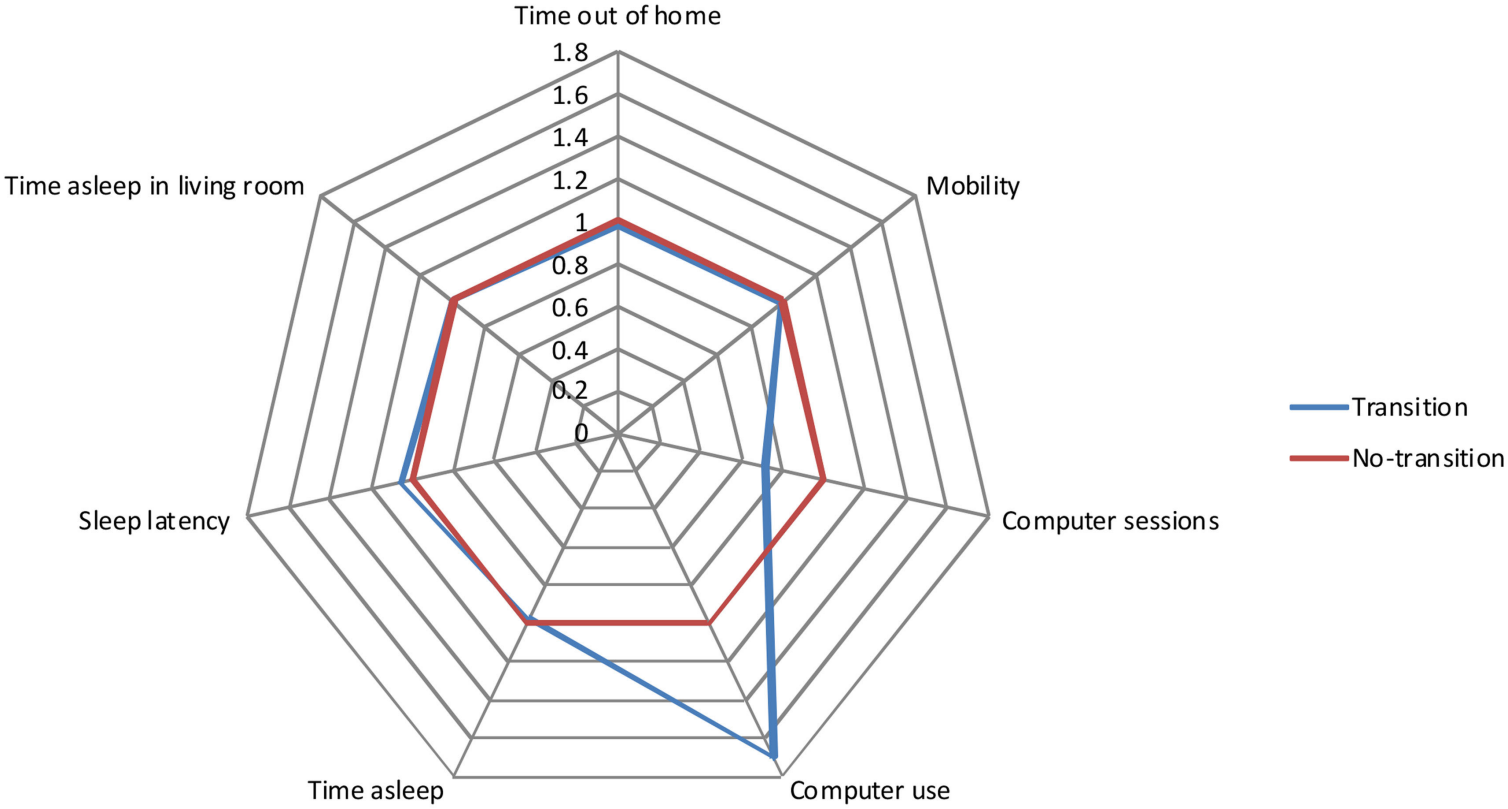
**Spider plot comparing the change in the odds ratio of transitioning (blue) compared to not transitioning (red) for several in-home measured variables**. This shows that computer use and sleep are especially important in predicting an advanced care transition.

**Figure 3:**
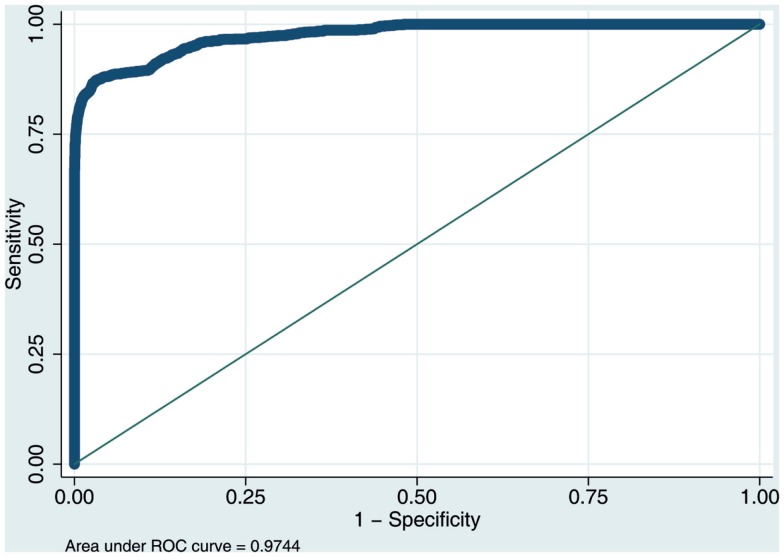
**ROC curve for a subset of 1,000,000 randomly selected data points comparing the trade-off between sensitivity and specificity of the logistic regression model’s performance for predicting transitions to a higher level of care**.

The results indicated that transitions to an advanced level of care were highly predictable and the model was a good fit for the data (McFadden’s *R*^2^ = 0.71; *p* < 0.00001). There was a clear pattern of in-home measured activity and behavior – a *behavioral signature* – associated with increased risk of transition to higher care. Figure 2 illustrates this graphically with a spider plot and Figure 3 shows the receiver operating curve (ROC). From Figure 2, it can be seen that in-home measured computer use and sleep latency are especially important for predicting whether an individual is likely to need additional care. In a sensitivity analysis, the area under the curve of the ROC was 0.947. These results are somewhat optimistic because no unseen data were used to test out-of-sample model predictions. However, the expected modest reduction in performance due to out-of-sample generalization would still allow precise predictions of who is likely to need additional care. Overall, these results suggest that fusing data collected in-home with more traditional assessments has the potential to improve diagnostic precision and efficiency. Future work will compare the predictive accuracy of different measurement domains (e.g., in-home assessment, clinical assessment, self-report, etc.) and to identify the optimally fused model that has the fewest and most predictive variables.

## Perceptions and Acceptance of Continuous In-Home Monitoring

ORCATECH research has been guided by the principal that technologies, which are not overly obtrusive or threatening to an individual’s sense of privacy or security, have a greater chance to be widely adopted by volunteers. In a focus group study of potential volunteers, acceptance of the in-home monitoring system was tied closely to perceived utility of the data gathered. If volunteers felt that their data would be useful in promoting research, they were more accepting of the in-home monitoring technology. Privacy was less of a concern to participants than anticipated (Wild et al., 2012). These results were reinforced in a survey of volunteers during the initial year of living with ORCATECH technology platform installed in their homes; almost 75% reported acceptance of in-home monitoring. However, in this initial survey, concerns for privacy and security were shared among more than half (60%) of those surveyed and these concerns increased after 1 year of participation. Nevertheless, participants were generally accepting of in-home monitoring, balancing their concerns over privacy and security with their desire to contribute to research that could potentially assist older individuals maintain independence (Boise et al., 2013).

## Limitations

To date ORCATECH’s data collection has predominantly employed wireless passive IR motion sensors, contact sensors, bed pressure sensors, and some non-content specific quantitative measures of computer use (typing speed, mouse use, duration of use, etc.). One limitation of using largely passive IR and contact sensors is the difficulty of distinguishing between individuals if there is more than one person in the home. In a recently published study, ORCATECH researchers explored using Gaussian mixture models (GMMs) combined with infrequent clinical assessments of gait velocity to model in-home walking speeds of two or more residents. These researchers were able to show that if the clinically measured gait velocities of residents are separated by at least 15 cm/s, a GMM can be accurately fit to the in-home gait velocity data (Austin et al., 2011a).

Other groups have experimented with solving the problem of user identification in a “smart home” using data approaches or augmenting the home or residents with additional devices. For example, Crandall and Cook (2011) proposed an algorithmic approach for tracking data from multiple residents in a single home. Srinivasan et al. (2010) used a height sensor installed in the home for resident identification and other groups have used wearables or video cameras (Wang et al., 2011; Banerjee et al., 2012; Ferdous et al., 2012). ORCATECH researchers are exploring using wearable technologies in several pilot studies. One such pilot study involves recording conversations during a specified segment of time. This requires participants to wear a digital recorder. Until it is possible to reliably distinguish between individuals in multiple person homes, one approach we have taken when analyzing our data is to limit our analyses to single-person homes only.

Another limitation is the low fidelity of recognizing and distinguishing between activities recorded. Currently, the data collected cannot distinguish between when someone is in the kitchen making a pot of coffee, unloading the dishwasher or surfing the internet on an ipad. Again there is a balance being drawn between respecting volunteer privacy and data collection.

Finally, “smart home” data collection can provide a wealth of information on what goes on in the home. Research would benefit from information that could be gathered on activities external for the home. With this in mind, ORCATECH researchers are currently exploring to expand data collection outside the home in tracking driving behavior and patterns and use of smartphones.

## Conclusion

Traditionally, individuals with increasing cognitive impairment experience functional decline. As individuals’ cognition becomes more impaired, they exhibit multiple behavioral changes, for example, difficulties in performing complicated tasks, limitations in engaging in meaningful social interactions, and challenges in maintaining mobility and efficiency of motor function. During a clinic visit, clinicians are only able to screen or “spot-check” changes in these functions during the short time period that the patient is in the examining room.

Continuous unobtrusive monitoring using pervasive in-home technology has allowed the capture of a wealth of data on individual daily activities previously not possible. As we and others continue to collect data and expand the participant pools, we are iteratively refining our understanding of how daily activities such as changes in night-time behaviors, mobility, computer use, medication adherence, and social engagement may track cognitive and functional changes and predict future cognitive impairment. This approach has clear application as an objective and sensitive tool for assessment in clinical trials. Given the density and frequency of the data, the precision of the estimates of change is orders of magnitude higher than current approaches. The possibility to collect a wealth of data through continuous monitoring reduces needed sample sizes and duration of studies and provides intra-individual predictions of change, while directly embodying the principle of personalized medicine.

While ORCATECH’s model applies widely to health assessment and intervention, it fits particularly well for meeting the current public health challenge posed by Alzheimer’s disease and related disorders. Home-based monitoring, in its ability to assess meaningful change, provides a method for tackling one of the greatest challenges across the spectrum of dementing diseases from pre-symptomatic to manifest disease.

Unobtrusive continuous in-home monitoring has the potential for revolutionizing how we conduct research in medicine and transforming clinical practice. The hope is that the wide adoption of continuous in-home monitoring will provide physicians and patients with the tools to predict and detect changes in health as they begin to occur – prior to detection with current tools. This would enable intervention to improve health outcomes, ultimately reducing the emotional and financial costs of disease.

## Conflict of Interest Statement

The authors declare that the research was conducted in the absence of any commercial or financial relationships that could be construed as a potential conflict of interest.
