# Corrigendum: Pervasive computing technologies to continuously assess Alzheimer's disease progression and intervention efficacy

**DOI:** 10.3389/fnagi.2015.00232

**Published:** 2015-12-11

**Authors:** Bayard E. Lyons, Daniel Austin, Adriana Seelye, Johanna Petersen, Jonathan Yeargers, Thomas Riley, Nicole Sharma, Nora Mattek, Hiroko Dodge, Katherine Wild, Jeffrey A. Kaye

**Affiliations:** ^1^Oregon Center for Aging and Technology, Oregon Health and Science UniversityPortland, OR, USA; ^2^Department of Neurology, Oregon Health and Science UniversityPortland, OR, USA; ^3^Department of Biomedical Engineering, Oregon Health and Science UniversityPortland, OR, USA; ^4^Neurology Service, Portland Veteran Affairs Medical CenterPortland, OR, USA

**Keywords:** Alzheimer's disease (AD), dementia, mild cognitive impairment (MCI), home monitoring, OHSU, ORCATECH, Layton Aging and Alzheimer's Disease Center

## Conflict of interest statement

The authors declare that the research was conducted in the absence of any commercial or financial relationships that could be construed as a potential conflict of interest.

